# Supportive care needs and service use during palliative care in family caregivers of patients with advanced cancer: a prospective longitudinal study

**DOI:** 10.1007/s00520-020-05565-z

**Published:** 2020-07-06

**Authors:** Anneke Ullrich, Gabriella Marx, Corinna Bergelt, Gesine Benze, Youyou Zhang, Feline Wowretzko, Julia Heine, Lisa-Marie Dickel, Friedemann Nauck, Carsten Bokemeyer, Karin Oechsle

**Affiliations:** 1 Palliative Care Unit, Department of Oncology, Hematology and BMT, University Medical Center Hamburg-Eppendorf, Martinistr, 52 20246 Hamburg, Germany; 2 Department of Medical Psychology, University Medical Center Hamburg-Eppendorf, Hamburg, Germany; 3 Department of Palliative Medicine, University Medical Center Goettingen, Goettingen, Germany; 4 Department of General Practice/Primary Care, University Medical Center Hamburg-Eppendorf, Hamburg, Germany

**Keywords:** Family caregiver, Cancer, Palliative care, Needs, Support services, Prospective studies

## Abstract

**Purpose:**

This study aimed to investigate the supportive care needs of family caregivers (FCs) of advanced cancer patients and their support service use at the beginning of specialist inpatient palliative care (SIPC), near the patient’s death, and during bereavement.

**Methods:**

FCs reported their needs using the Family Inventory of Needs (FIN), along with their utilization of psychosocial and bereavement support services at the beginning (*N* = 232) and 6–9 months after SIPC (*N* = 160).

**Results:**

At the beginning of SIPC, mean of 16.9 of 20 needs were reported to be highly important, and 12.2 were reported to be met. At the time of the patient’s death, 16.8 needs were highly important, and 13.8 were met. At both time points, the highest ranked need was related to information about changes in the patient’s condition (100% vs. 99%), and the most frequently unmet need was related to feeling hope (73% vs. 71%). Multivariate linear regression analysis revealed a low education level to be consistently related to a greater number of highly important needs. Higher satisfaction with care and better social support was related to a greater number of met needs. Twenty-five percent of FCs had accessed at least one psychosocial support service prior to SIPC, and 30% had done so during bereavement. Among non-users of support services, > 75% indicated sufficient informal support as a barrier to service use.

**Conclusions:**

The findings offer a useful guide for adequately addressing FCs’ needs in an effort to optimize FC support. However, only a subgroup of the FCs used support services. Better information and provision of tailored services might improve FCs’ situations in the future.

## Introduction

Family caregivers (FCs) support patients with advanced cancer but are also affected by the patient’s disease themselves and experience specific but often unmet supportive care needs. In a current review, the most commonly identified unmet needs for informal caregivers were information needs, including illness and treatment information (26–100%) and care-related information (21–100%) [1]. Additional frequently reported unmet FC needs were education in symptom management and care [2–5], daily living needs [6, 7], dealing with prognostic uncertainty [7–10], and reducing the patient’s stress [7, 11]. Fringer et al. suggested maintaining normality during transitions to be the central need of patients and their FCs [12]. The main categories of FC needs were found to be social, cognitive, and psychological needs [13] as well as knowledge and competence, preparedness, and role confidence [14]. While the number of needs seem to remain stable over time, specific needs seem to be time dependent: Managing difficult aspects of the patient’s behavior and adjusting to changes in the patient’s personality seem to increase over time [7], while the “need for knowledge about the disease” and other information needs seem to decrease over time [9]. Studies showed that after the patient’s death, FCs claimed that their own needs had not been adequately addressed during the patient’s disease and that support was insufficient [15, 16]. Bereaved FCs also reported a lack of bereavement support and emotional assistance [16] or impersonal, generic, or just standard practice bereavement support [15].

Higher numbers of unmet needs were associated with psychological distress in FCs [7, 11] and overall FC burden [2, 17, 18]. Further need-related factors with a negative impact on FCs’ burden were a lack of care training, low available support, a large discrepancy between FCs’ and patient’s reports of patient’s unmet need, as well as assistance managing medical, non-medical care and direct patient care activities [4, 17, 19]. Overall, the impact of FCs’ sociodemographic characteristics on their supportive needs seems to be low according to the current literature [11], but there might be some effects of partnership and financial aspects [20, 21]. In contrast, satisfaction with patient care seems to be important for the perception of one’s own unmet needs in FC [2, 22]. Studies have suggested that FCs’ needs might be more likely to be met when the patient is treated in a specialist palliative care setting [23], and some studies have distinguished specific aspects contributing to potential benefits as well as relevant deficits [24–28]. However, the impact of palliative care on FCs’ needs remains controversial [29].

Support service utilization in FC has rarely been investigated, but Dionne-Odom et al. reported a utilization rate of approximately one-third [30]. In this study, depressive symptoms, anxiety, and preparedness were associated with utilization and FCs’ interest in such services [30]. Interest in services also seemed to depend on the number of supportive care needs [11], minority status, shorter duration of caregiving, and higher stress burden [30]. However, Aoun et al. found that the most frequently used sources of support for FC were informal sources, such as family, friends, and funeral providers. Professional sources were the least used and were related to the highest proportions of perceived unhelpfulness, whereas unhelpfulness was perceived lower for informal support [31].

Overall, data on the needs of FCs caring for an advanced-cancer patient in the SIPC setting, factors associated with need profiles, and the utilization of support services are rare and heterogeneous. Therefore, the aims of this multicenter study were to prospectively investigate (1) the importance and satisfaction of needs among FCs of patients with advanced cancer at the beginning of specialist inpatient palliative care (SIPC), near the patient’s death and during bereavement; (2) factors associated with the amount of important and met needs; and (3) FCs’ utilization of psychosocial support and bereavement services.

## Methods

### Sample and design

Between June 2016 and June 2017, FCs were consecutively recruited in two university medical centers within 72 h after the advanced cancer patient’s first admission to the SIPC ward. Inclusion criteria included being the primary informal caregiver as indicated by the patient and being over the age of 18. The exclusion criteria were imminent death, legal guardianship, and inadequate language skills or cognitive impairment that would interfere with giving fully informed consent and completing questionnaires.

Data were collected at two time points by self-report questionnaires. The FC situation at the beginning of SIPC was assessed within 72 h after admission (T1), and non-respondents were personally reminded after 2 working days. The second assessment was performed 6 months after the patient’s discharge from or death on the SIPC ward (T2). Among the FCs of patients who were alive after 6 months, the second assessment was postponed up to a maximum of 9 months. Non-respondents received a written reminder after approximately 4 weeks.

### Instruments

#### Outcome variables (T1 and T2)

##### Supportive care needs

To assess needs at the beginning of SIPC (T1) and needs experienced within the last 7 days of the patient’s life (retrospective rating, T2), FCs completed a German version of the 20-item Family Inventory of Needs (FIN) [32, 33]. On the FIN, needs are rated on two subscales, FIN-Importance (1 “not important” to 5 “extremely important”) and FIN-Fulfillment (0 “not met,” 0.5 “partly met,” and 1 “met”), with the score for the latter subscale only calculated for needs indicated to be at least “somewhat important.” To estimate needs related to preparedness and psychosocial assistance, 7 items were added to the FIN questionnaire.

##### Utilization of support services

FCs reported their use of sources of information and support prior to SIPC (assessed at T1) and after the patient’s death (assessed at T2), their perceived helpfulness of accessed services, and barriers to using psychosocial support services. Additionally, data on the utilization of bereavement care provided by the two study wards, which included open bereavement cafés, commemoration ceremonies, and counseling on external, locally available bereavement care, were collected (assessed at T2).

#### Baseline factors potentially related to supportive care needs (T1)

##### Subjective psychological distress

The Distress Thermometer (DT) is an analogue scale rated from 0 “no distress” to 10 “extreme distress,” with values ≥ 5 indicating a relevant burden with the need for professional psychosocial support [34]. The DT was validated for distress screening in FCs in a previous study, which indicated the same cut-off value of ≥ 5 [35]. In addition to the DT, an adapted problem list including assessment of physical strain was administered [36].

##### Depressive and anxiety symptoms

FCs completed the Patient Health Questionnaire-Depression module (PHQ-9 [37]) and the General Anxiety Disorder Scale (GAD-7 [38]), which have total scores ranging from 0 to 27 and 0 to 21, respectively.

##### Palliative care outcome

FCs’ multidimensional perspectives on the patient’s situation were assessed by a caregiver-adapted version of the 7-day recall staff version of the Integrated Palliative Care Outcome Scale (IPOS) [39], which has a total score ranging from 0 to 68.

##### Satisfaction with care

Satisfaction with team-based services, including symptom relief, information, FC support, and patient psychosocial care, was assessed by using the Family Carer Satisfaction with Palliative Care scale (FAMCARE-2) [40, 41], which has a total score ranging from 17 to 85.

For the DT, GAD-7, PHQ-9, and IPOS, higher values indicate worse outcomes. For the FAMCARE-2, a higher score reflects higher satisfaction.

##### Sociodemographic-, medical-, and care-related variables

FCs reported their own and the patient’s sociodemographic characteristics; their relationship to the patient; and information relating to the patient’s diagnosis, care, and death. FCs’ socioeconomic status was assessed using a composite indicator score [42], and migrant background was assessed using a basic set of indicators for mapping migrant status [43]. FCs’ social support was measured by the Oslo-3-Item-Social-Support Scale (OSLO-3). The total score ranges from 3 to 14, with categorization into poor (3–8), moderate (9–11), and strong (12–14) support [44, 45].

### Statistical analysis

Descriptive statistics were calculated, including the frequency distributions, percentages, means, and standard deviations.

For both time points, explorative multivariate linear regression analyses using backwards variable selection procedures were conducted, with the number of very/extremely important needs and the number of needs met being the dependent variables. In the regression analyses, the same set of independent baseline variables (measured at T1) were included at step 1: FC characteristics (age, gender, relationship to the patient, religious confession, education, socioeconomic status, work status, and social support), patient- and care-related variables (patient age, time since diagnosis, prior care site, and FCs’ prior involvement in caregiving), prior use of psychosocial support services, satisfaction with care, and palliative care outcome as well as FCs’ physical and mental strain (exhaustion, sleep disturbances, distress, and depressive and anxiety symptoms). Categorical variables were dichotomized. The examination of correlations among independent variables revealed no problems with multicollinearity, except for working situation and FCs’ age and depressive and anxiety symptoms. Thus, working situation and depressive symptoms were omitted from further analysis. Missing data were handled using the listwise deletion method.

All significance tests were two-sided using a significance level of *α* < 0.05. All analyses were performed using the statistical package SPSS version 24.0 (IBM, USA).

## Results

### Sample recruitment and characteristics

#### Recruitment procedures

**Fig. 1:**
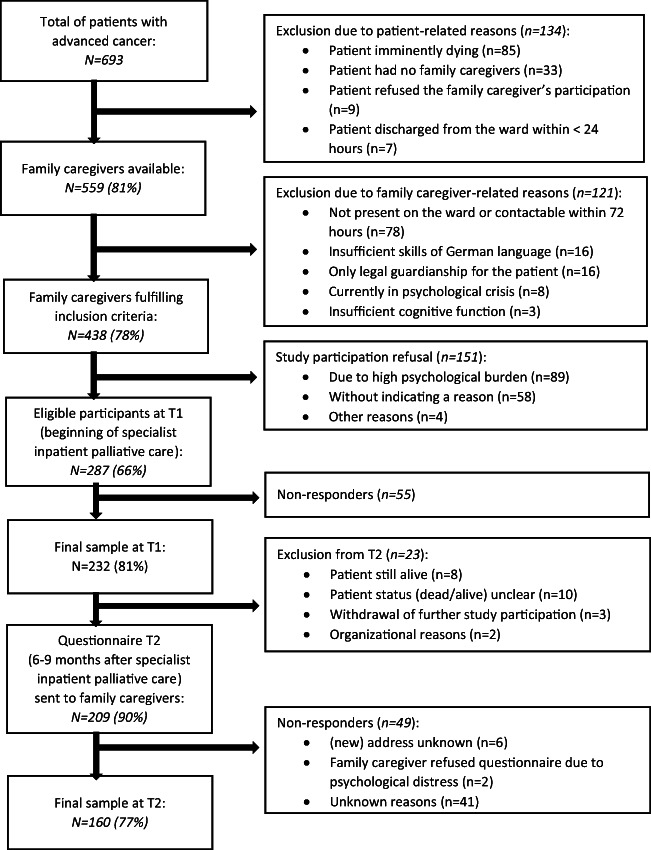
Study recruiting process and sample development

A total of 438 FCs met the inclusion criteria. Of these FCs, 287 agreed to participate (66%), and 232 (response rate: 81%) returned the first questionnaires (T1). At follow-up (T2), 160 FCs returned the second questionnaire (response rate: 77%). Details are presented in Fig. 1. The mean time between T1 and T2 was 6.7 months.

#### Non-respondent analyses

Comparing respondents to non-respondents, we did not find significant differences in sociodemographic characteristics (age, gender, marital status, educational level, working situation, socioeconomic status, and relationship to the patient assessed at T1; *p* = 0.058 to 0.854) or in psychological burden (distress, anxiety symptoms, and depressive symptoms at T1; *p* = 0.358 to 0.863).

#### Sample characteristics

The mean age of the 232 FCs was 55.5 ± 14.8 years (T1); female FCs constituted 66% of the sample, and spouses/partners constituted 64%. The time since the patient’s diagnosis was ≤ 12 months in 43% of cases (Table 1). At the second assessment (T2), the mean time since patient death was 6.0 months (SD 1.4; range, 1–9), and 96 out of 160 patients (60%) had died at the SIPC ward (not shown).

**Table 1:** Sample characteristics and family caregivers mental burden, palliative care outcome and satisfaction with care at the beginning of SIPC (T1; *N* = 232)

*Family caregiver characteristics*		
Age, M (SD); range		55.5 (14.8); 20–88
Relationship to the patient. Patient is…, *n* (%)	Spouse/partner	148 (63.8)
	Mother/father	61 (26.3)
	Others ^a^	23 (9.9)
Marital status, *n* (%)	Single	36 (15.5)
	Married or life partnership	164 (70.7)
	Divorced or widowed	29 (12.5)
	Missing	3 (1.3)
Having Children, *n* (%)	Yes	164 (70.7)
	No	24 (27.6)
	Missing	4 (1.7)
Religious confession, *n* (%)	Yes	153 (65.9)
	No	75 (32.3)
	Missing	4 (1.7)
Migrant background, *n* (%)	None	212 (91.4)
	First generation	15 (6.5)
	Second generation	5 (2.2)
Educational level, *n* (%)	Elementary school (≤ 9 years)	65 (28.0)
	Junior high school (10 years)	72 (31.0)
	High school (12–13 years)	91 (39.2)
	Missing	4 (1.7)
Current working situation, *n* (%)	Working	123 (53.0)
	Not working	99 (42.7)
	Missing	10 (4.3)
Socioeconomic status, *n* (%)	Low	44 (19.0)
	Moderate	111 (47.8)
	High	73 (31.5)
	Missing	4 (1.7)
Perceived social support (OSLO-3), M (SD); range		10.7 (2.3); 4–14
Perceived social support categories (OSLO-3), *n* (%)	Poor	43 (18.5)
	Moderate	90 (38.8)
	Strong	98 (42.2)
	Missing	1 (0.4)
*Patient- and care-related characteristics*		
Patient’s gender, *n* (%)	Male	118 (50.9)
	Female	105 (45.3)
	Missing	9 (3.9)
Patient age, *n* (%)	≤ 60 years	75 (32.3)
	> 60 years	155 (66.8)
	Missing	2 (0.9)
Time from patient‘s diagnosis, *n* (%)	≤ 12 months	99 (42.7)
	> 12 months	125 (53.9)
	Missing	8 (3.4)
Prior care site, *n* (%)	At home without any nursing service	82 (35.3)
	At home with nursing service	21 (9.1)
	At home with specialist outpatient palliative care service	33 (14.2)
	Hospital inpatient ward	82 (35.3)
	Others ^b^	11 (4.8)
	Missing	3 (1.3)
Existence of a patient decree, *n* (%)	Yes	140 (60.3)
	No	92 (39.7)
Existence of a power of attorney ^c^, n (%)	Yes	159 (68.5)
	No	73 (31.5)
Prior involvement of the family caregiver in patient care, *n* (%)	Yes	107 (46.1)
	No	118 (50.9)
	Missing	7 (3.0)
*Family caregivers physical and mental burden*		
Exhaustion, *n* (%)	Yes	171 (73.7)
	No	51 (22.0)
	Missing	10 (4.3)
Sleep disturbances, *n* (%)	Yes	163 (70.3)
	No	60 (25.9)
	Missing	9 (3.9)
Distress (DT), M (SD); range		7.9 (1.8); 0–10
Anxiety symptoms (GAD-7), M (SD); RANGE		9.4 (5.1); 0–21
Depressive symptoms (PHQ-9), M (SD); Range		9.0 (5.7); 0–27
*Family caregivers assessment of palliative care*		
Palliative care outcome (IPOS), M (SD); Range		37.9 (7.7); 12–58
Satisfaction with care (FAMCARE-2), M (SD); range		73.7 (9.6); 44–85

*M*, Mean; *SD*, standard deviation; *FC*, family caregiver; *SIPC*, specialist inpatient palliative care; *OSLO-3*, OSLO-3-Item-Social-Support Scale; *DT*, Distress Thermometer; *GAD-7*, General Anxiety Disorder Scale; *PHQ-9*, Patient Health Questionnaire-Depression module; *IPOS*, Integrated Palliative Outcome Scale; *FAMCARE-2*, Family Caregiver Satisfaction with Palliative Care scale

^a^Adult children, siblings, close friends or other relatives; ^b^ Nursing home in 9 patients or other care facilities; ^c^ The patient had appointed the family caregiver to act as substitute decision-maker in terms of personal (including health) matters. Thus, the family caregiver was permitted under the law to make decisions on behalf of the patient regarding medical decisions, if the patient lacked decision-making capacity

### Importance and fulfillment of supportive care needs among family caregivers

#### Beginning of SIPC (T1)

FCs rated a mean number of 16.9 of 20 needs (85%) to be very/extremely important (FIN-Importance; SD 2.6; range, 8–20). The highest ranked need was related to information about changes in the patient’s condition (100%), and the lowest ranked need concerned the FC’s own welfare (27%). The mean number of met needs was 12.2 of 20 (FIN-Fulfillment; SD 5.4, range 0–20), representing 60% of needs (not shown). However, seven needs were unmet in more than 50% of participants, with needs related to hope (73%) and knowing when to expect symptoms (63%) being the most common unmet needs (Table 2).

**Table 2:** Importance and fulfillment of family caregiver needs at the beginning of SIPC (*N* = 232) and within the last 7 days of the patient’s life (*N* = 160)

	At the beginning of SIPC (T1)(*N* = 232)				Within the last 7 days of the patient’s life (T2) (retrospective assessment, *N* = 160)			
*FIN—family inventory of needs; needs are ranked in order of very/extreme importance* *I need/needed to…*		*Importance*		*Fulfillment*	*Importance*		*Fulfillment*	
	M (SD)	“Very or extremely important” n/N (%)	Rank	“Not or only partly met”^a^ n/N (%)	M (SD)	“Very or extremely important” n/N (%)	Rank	“Not or only partly met”^a^ n/N (%)
…be informed of changes in the patient’s condition	4.7 (0.5)	228/229 (99.6)	1	15/206 (7.3)	4.6 (0.5)	157/158 (99.4)	1	51/154 (33.1)
…feel that the professionals care about the patient	4.5 (0.8)	226/230 (98.3)	2	3/215 (1.4)	4.8 (0.5)	158/159 (99.4)	1	14/155 (9.0)
...be assured that the best possible care is given to the patient	4.7 (0.6)	225/229 (98.3)	2	68/209 (32.5)	4.7 (0.6)	150/156 (96.2)	3	36/150 (24.0)
…have my questions answered honestly	4.5 (0.6)	221/229 (96.5)	3	89/216 (41.2)	4.5 (0.6)	154/159 (96.9)	2	64/156 (41.0)
…know exactly what is being done to the patient	4.5 (0.6)	218/229 (95.2)	4	73/210 (34.8)	4.5 (0.6)	152/158 (96.2)	3	51/156 (32.7)
...have information given in terms that are understandable	4.6 (0.7)	217/230 (94.3)	5	54/206 (26.2)	4.5 (0.7)	150/158 (94.9)	4	34/152 (22.4)
…know why things are done for the patient	4.5 (0.7)	212/226 (93.8)	6	81/200 (40.5)	4.4 (0.7)	140/153 (91.5)	6	51/147 (34.7)
...know the probable outcome of the patient’s illness	4.5 (0.8)	208/225 (92.4)	7	110/200 (55.0)	4.4 (0.8)	141/154 (91.6)	5	64/145 (44.1)
...know what symptoms the treatment or disease can cause	4.4 (0.7)	206/224 (92.0)	8	35/199 (17.6)	4.4 (0.8)	142/154 (92.2)	8	73/148 (49.3)
…be told about changes in treatment plans while they are being made	4.4 (0.7)	205/225 (91.1)	9	82/199 (41.2)	4.4 (0.7)	143/154 (92.9)	7	57/149 (38.4)
…know specific facts concerning the patient’s prognosis	4.4 (0.9)	200/225 (88.9)	10	104/206 (50.5)	4.4 (0.8)	143/158 (90.5)	9	71/152 (46.7)
…have information about what to do for the patient at home	4.4 (1.1)	184/209 (88.0)	11	89/166 (53.6)	4.1 (1.3)	108/132 (81.8)	12	43/109 (39.4)
…know when to expect symptoms to occur	4.3 (0.9)	196/225 (87.1)	12	122/194 (62.9)	4.1 (1.0)	123/153 (80.4)	13	78/141 (55.3)
…know what treatment the patient is receiving	4.4 (0.8)	197/228 (86.4)	13	69/207 (33.3)	4.3 (0.8)	131/157 (83.4)	10	48/152 (31.6)
…feel there is hope	4.4 (1.1)	167/207 (80.7)	14	126/173 (72.8)	4.0 (1.3)	104/138 (75.4)	14	82/116 (70.7)
…feel accepted by the health professionals	4.1 (1.0)	173/226 (76.5)	15	27/196 (13.8)	4.2 (0.8)	128/154 (83.1)	11	23/147 (15.6)
…be told about people who could help with my problems	3.8 (1.1)	152/221 (68.8)	16	102/187 (54.5)	3.6 (1.1)	93/149 (62.4)	17	56/137 (40.9)
…help with the patient’s care	3.8 (1.2)	144/224 (64.3)	17	45/176 (25.6)	3.8 (1.3)	104/153 (68.0)	15	17/129 (13.2)
…know the names of the health professionals involved in the patient’s care	3.7 (1.0)	138/229 (60.3)	18	88/197 (44.7)	3.8 (1.0)	102/154 (66.2)	16	30/144 (20.8)
…have someone be concerned with my health	2.6 (1.3)	59/221 (26.7)	19	85/147 (57.8)	2.8 (1.3)	47/151 (31.1)	18	59/112 (52.7)
*Additional needs to FIN (study-specific)*								
…be prepared for death and bereavement	3.6 (1.3)	128/215 (59.5)	n.a.	118/168 (70.2)	3.5 (1.4)	84/148 (56.8)	n.a.	73/127 (57.5)
…have more information about alternative/complementary therapy	3.1 (1.5)	104/215 (48.4)	n.a.	117/141 (83.0)	3.0 (1.5)	59/140 (42.1)	n.a.	78/101 (77.2)
…get counseling with focus on social aspects/further patient care	4.3 (1.0)	185/218 (84.9)	n.a.	120/181 (66.3)	3.6 (1.4)	94/144 (65.3)	n.a.	58/119 (48.7)
…get psychological care	2.8 (1.4)	78/217 (35.9)	n.a.	88/123 (71.5)	3.0 (1.5)	61/146 (41.8)	n.a.	60/104 (57.7)
…get counseling with focus on children/family	2.4 (1.5)	64/216 (29.6)	n.a.	71/97 (73.2)	2.5 (1.5)	46/138 (33.3)	n.a.	42/76 (55.3)
…get spiritual/pastoral care	2.2 (1.3)	43/221 (19.5)	n.a.	73/99 (73.7)	2.2 (1.4)	38/144 (26.4)	n.a.	39/69 (56.5)
…get music- or art-therapy	1.6 (1.0)	18/222 (8.1)	n.a.	42/59 (71.2)	1.8 (1.2)	19/141 (13.5)	n.a.	32/50 (64.0)

*SIPC*, specialist inpatient palliative care; *FIN*, Family Inventory of Needs: ; n.a., not applicable

^a^ Only calculated if the need was reported to be at least „somewhat important “at the respective point of time

#### Last 7 days of the patient’s life (T2)

FCs retrospectively rated a mean number of 16.8 needs to be very/extremely important (FIN-Importance; SD 3.1; range, 7–20), representing 85% of needs (not shown). Again, the need that was indicated to be most important was being informed about changes in the patient’s condition (99%), and the least important was related to the FC’s own welfare (31%). Overall, a mean number of 13.8 needs were met (FIN-Fulfillment; SD 4.8, range 3–20), representing 70% of needs. Three needs remained unmet in more than 50% of participants. Again, needs related to hope (71%) and knowing when to expect symptoms (55%) were most frequently unmet (Table 2).

### Baseline factors associated with numbers of highly important and met needs

#### Beginning of SIPC (T1)

In order of magnitude, FC’s lower education, FC’s older age, patient’s home-based care prior to SIPC, and patient’s higher distress were associated with a higher number of very/extremely important needs (FIN-Importance; *ß* = 0.148 to − 0.235; *p* = 0.004 to 0.043). Factors associated with higher numbers of met needs were FC’s higher satisfaction with care, FC not being appointed as a substitute decision-maker, FC’s stronger social support, patient’s diagnosis ≤ 1 year prior to SIPC, and FC’s better assessment of the palliative care outcome (FIN-Fulfillment; *ß* = − 0.120 to 0.580; *p* < 0.001 to 0.037). The final models accounted for 25% of the variance in the number of important needs and 53% of the variance in the number of met needs (Table 3).

**Table 4:** Family caregivers utilization of support services prior SIPC (T1; *N* = 232) and after the patient’s death (T2; *N* = 160)

	Prior to SIPC (T1) (N = 232)	After the patient’s death (T2) (N = 160)
	n/N (%)	n/N (%)
Utilization of ≥1 support service, n (%) yes ^a^	58/232 (25.0%)	48/160 (30.0)
Support services used (multiple answers possible), n (%) yes		
Psychological counseling	38/232 (16.4)	17/160 (10.6)
Social counseling	15/232 (6.5)	14/160 (8.8)
Spiritual counseling	15/232 (6.5)	12/160 (7.5)
Counseling on parenting/family issues	10/232 (4.3)	2/160 (1.3)
Counseling at cancer information center	13/232 (5.6)	4/160 (2.5)
Self-help group	8/232 (3.4)	9/160 (5.6)
Bereavement care ^b^	n.a.	18/160 (11.3)
Utilization of ≥1 bereavement support provided by the study wards, n (%) yes	n.a.	46/127 (36.2)
Study wards’ bereavement support used (multiple answers possible), n (%) yes ^c^		
Commemoration ceremony	n.a.	25/119 (21.0)
Open bereavement café	n.a.	12/116 (10.3)
Counseling for locally available, external bereavement care	n.a.	6/114 (8.3)
Barriers for accessing support services in non-users (multiple answers possible), n (%) yes ^d^		
Sufficient informal support	129/152 (84.9)	73/94 (77.7)
No subjective need	107/150 (71.3)	55/93 (59.1)
Lack of time	64/142 (45.1)	24/87 (27.6)
Preferring support by treating physicians	65/143 (45.5)	28/88 (31.8)
No expectation of subjective benefit	49/134 (36.6)	39/85 (45.9)
Lack of knowledge about psychosocial services	50/146 (34.2)	27/90 (30.0)
Services too far away	28/139 (20.1)	20/85 (23.5)
Potential burden to family/partnership	5/142 (3.5)	9/88 (10.2)

Abbreviations: SIPC, specialist inpatient palliative care; n.a., not applicable

^a^ Including external bereavement services and those of the study wards after the patient’s death; ^b^ Only external bereavement services; ^c^ Services only provided at one study ward are not displayed; ^d^ Only family caregivers who reported not having accessed any service at respective times were included (T1: *N* = 174; T2: *N* = 112)

#### Last 7 days of the patient’s life (T2)

In order of magnitude, FC’s lower education, FC’s low socioeconomic status, FC’s lack of utilization of support services prior to SIPC, and patient’s diagnosis ≤ 1 year prior to SIPC were related to a higher number of very/extremely important needs (FIN-Importance; *ß* = 0.186 to − 0.321; *p* = 0.002 to 0.045). Factors associated with a higher number of met needs were FC’s higher care satisfaction, FC’s stronger social support, and FC not being appointed as a substitute decision-maker (FIN-Fulfillment; *ß* = − 0.222 to 0.343; *p* = 0.005 to 0.032). The final models accounted for 38% of the variance in the number of important needs and 35% of the variance in the number of met needs (Table 3).

### Use of psychosocial and bereavement support services

#### Use of psychosocial support services prior to SIPC (T1)

Prior to the patient’s admission to the SIPC ward, 25% of FCs had used at least one source of information or support. The most frequently accessed service was psychological counseling, which was used by 16% of FCs. Overall, 58 FCs had accessed 108 services, which were rated “helpful” (vs. not) in 60% of cases. Among 174 non-users, the strongest barrier was sufficient informal support (85%; Table 4).

**Table 3:** Factors associated with family caregiver needs at admission to SIPC (T1; N = 232) and within the last 7 days of the patient’s life (T2; N = 160)

	At the beginning of SIPC (N = 232) ^a,b^				Within the last 7 days of the patient’s life (retrospective assessment, *N* = 160) ^c,d^			
	b	S.E. b	ß	p	b	S.E. b	ß	p
**Regression models for number of very/extremely important needs** ^**e**^								
FC’s age	.036	.014	.204	.012*	‡			
FC’s education [up to junior high school = 0; high school = 1]	-.1.154	.391	−.235	.004*	−2.021	.621	−.321	.002*
FC’s socioeconomic status [moderate/high = 0; low =1]]	‡				2.231	.806	.272	.007*
Relationship to the patient [others = 0; spouse/partner = 1]					1.087	.606	.173	**.077**
Time since patient’s cancer diagnosis prior to SIPC [>1 year = 0; ≤1 year = 1]	‡				1.156	.567	.186	.045*
Care site prior to SIPC [others = 0; at home = 1]	.962	.379	.195	.012*	‡			
FC’s assessment of the palliative care outcome (IPOS)	‡				.074	.039	.167	.063
Utilization of support services prior to SIPC [yes = 0; no = 1]	‡				1.759	.673	.247	.011*
Distress (DT)	.262	.128	.173	.043*	‡			
Anxiety symptoms (GAD-7)	.070	.040	.148	.083	‡			
**Regression models for number of met needs** ^**e**^								
FC’s religious confession [no = 0; yes = 1]	−.1311	.761	−.120	.088	‡			
FC’s education [up to junior high school = 0; high school = 1]	‡				−2.300	1.156	−.216	.051
Time since patient’s cancer diagnosis prior to SIPC [>1 year = 0; ≤1 year = 1]	1.563	.721	.150	.033*	‡			
Existence of power of attorney [no = 0; yes = 1]	−1.980	.792	−.171	.014*	−2.705	1.237	−.222	.032*
Involvement in patient care prior to SIPC [no = 0; yes = 1]	‡				−1.828	1.060	−.176	.089
FC’s assessment of the palliative care outcome (IPOS)	−.100	.048	−.144	.037*	‡			
FC’s satisfaction with palliative care (FAMCARE-2)	.287	.035	.580	<.001*	.189	.057	.343	.001*
Perceived social support (OSLO-3)	.425	.175	.168	.017*	.706	.257	.286	.008*

Abbreviations: b, Beta; S.E. b, standardized error; ß, standardized b; *p* value, probability of type I error; SIPC, specialist inpatient palliative care; IPOS, Integrated Palliative Care Outcome Scale (higher values indicate worse palliative care outcome); FAMCARE-2, Family Caregiver Satisfaction with Palliative Care Scale (higher values indicate higher satisfaction); DT, Distress Thermometer (higher values indicate higher distress); OSLO-3, OSLO-3-Item-Social-Support Scale (higher values indicate stronger social support)

^a^ Number of very/extremely important needs at the beginning of SIPC: R^2^ = .254, F(5,129) = 8.773, *p* < .001; Tolerance values between .804–985

^b^Number of met needs at the beginning of SIPC: R^2^ = .527, F(6,102) = 18.964, p < .001; Tolerance values between .957–.991

^c^Number of very/extremely important needs within the last 7 days of the patient’s life: R^2^ = .378, F(6,80) = 8.103, p < .001; Tolerance values between .799–.984

^d^Number of met needs within the last 7 days of the patient’s life: R^2^ = .346, F(5,67) = 7.101, p < .001; Tolerance values between .828–.944

^e^All potential predictor variables were measured at the beginning of SIPC (T1)

‡ Not included in the final regression model due to stepwise backwards selection procedure

*All significant *p*-values are marked with an asterisks

#### Use of psychosocial and bereavement services after the patient’s death (T2)

During bereavement, 46 (36%) FCs reported using bereavement services provided by the two study wards, with commemoration ceremonies being the most commonly used service (21%). Among only the FCs of the 96 patients who had died on the study ward, approximately half used the wards’ bereavement services. Beyond these services, 30% of this subgroup of FCs had accessed at least one external source of information and support, with bereavement care (11%) and psychological counseling (11%) being the most frequent. In total, 48 FCs had accessed 87 support services, which were rated “helpful” in 90% of cases. Among 112 non-users, the most frequent barrier was sufficient informal support (78%; Table 4).

## Discussion

In this prospective multicenter study, FCs indicated a consistently high rate of approximately 85% of needs being very/extremely important (FIN-Importance) at the beginning of SIPC as well as at the time the patient was dying. Interestingly, the importance of specific FC needs did not change substantially over time; the following FC needs remained the top five needs over time: “being informed of changes in the patient’s condition,” “feeling that the health professionals care well for the patient,” “being assured the best possible care is being given to the patient,” “having my questions answered honestly,” and “knowing exactly what is being done to the patient.” In contrast, two studies with FCs of patients with neuro-oncological tumors suggested that the quality of FC needs changes during the patient’s disease trajectory [7, 9], which was not confirmed in our study. However, neither study evaluated FCs at a time when the patient was nearing death.

At the beginning of SIPC, the mean rate of met needs (FIN-Fulfillment) was 60%. A previous study described a comparable rate of met needs in 67% of FCs in a palliative care setting [23]. In our study, the mean percentage of met needs increased slightly to 70% at the time the patient was dying. This finding indicates that approximately 30–40% of needs were unmet from a longitudinal perspective, which seems alarming since the unsolved problems or unmet needs of FCs can negatively affect their own quality of life as well as patient health outcomes [46]. On the other hand, the number of needs unmet in more than 50% of FCs decreased from 7 to 3 out of 20 needs over time. Notably, the remaining three needs had already been unmet at the beginning of SIPC. These unmet needs were related to hope, knowledge of when to expect the occurrence of symptoms and the FC’s own welfare.

A comparison of the satisfaction of specific needs at the beginning of SIPC and at the time of the patient’s death showed that some needs changed in more than 15% of FCs, which might be interpreted as clinically relevant. The particularly affected needs were “being informed of changes in the patient’s condition” (7% unmet at beginning of SIPC vs. 33% at time of patient’s death) and “knowing what symptoms the treatment or disease can cause” (18% vs. 49%). When patients enter SIPC, information about the patient’s health status and cause of symptoms might be most explicitly and extensively discussed during the first clinical encounters with FCs. However, FCs do not receive such information later in the care process, as demonstrated by our results. In contrast, the rates of unmet needs decreased substantially with regard to having information about people who could help with FCs’ problems (55% vs. 41%) and knowing the names of the members palliative care team (45% vs. 21%). Several studies reported unmet needs of FCs during the patient’s disease, which primarily concerned information, symptom management and medication, patient care, and FC needs regarding day-to-day life in light of the patient’s disease [2–4, 6]. Additional studies evaluated FCs’ perspectives after the patient’s death retrospectively and indicated a lack of fulfillment of needs, with 30–50% of FCs complaining of a lack of interest in their emotional and psychological distress pre-bereavement, a lack of or little information about the prognosis or disease trajectory, or a lack of FC-directed support offers [15, 16]. In many aspects, our results support these findings but add the dimension of spiritual needs, as feeling hope was a consistently important and often unmet need in FCs over time. Albeit to a lesser extent, preparedness for death and bereavement, which could also be seen as spiritual need, was also indicated as important by a substantial number of FCs.

Our study aimed to investigate factors related to FC needs, both at the beginning of SIPC and at the time the patient was dying. Regarding sociodemographic, patient- and care-related factors, multivariate regression analyses revealed that a lower education level was consistently associated with the number of very/extremely important needs (FIN-Importance). However, a higher education level had no effect on the number of met needs (FIN-Fulfillment). A recent review showed varying results regarding the impact of educational level on unmet needs of FCs [1]. The FC not being appointed as a substitute decision-maker was a factor that was consistently associated with the number of met needs (FIN-Fulfillment). The latter finding suggests that FCs who are potentially involved as surrogates in forthcoming decisions regarding patient care might experience more unmet needs. To our knowledge, this association has not been investigated in the palliative care context. In contrast to other researchers [11, 21, 23], we found a relationship neither between FC needs and being a spouse/partner nor between FC needs and patient age. Beyond sociodemographic factors, higher satisfaction with care was associated with higher numbers of met needs (FIN-Fulfillment) at both points of time. Our results, together with those of a study by Fredriksdottir [23], demonstrate the capability of team-based, holistic SIPC to provide better need fulfillment than care in acute hospital wards [23]. Additionally, we found that stronger social support was related to a higher number of met needs (FIN-Fulfillment) at both points of time, underscoring the role of informal sources of support for meeting FCs’ needs, as suggested by Aoun et al. [31]. Thus, the availability of quality SIPC and informal social support seems beneficial for fulfilling needs of FCs. Among indicators of mental burden, distress was the only factor associated with more very/extremely important needs (FIN-Importance) at the beginning of SIPC. Previous studies have shown associations of distress and psychological burden with FC needs, but these effects might be predictive in both directions [7, 11, 17, 18].

Despite the high number of very/extremely important needs and needs reported to be unmet, the number of FCs who had used psychosocial support services was low, with 25% using services prior to SIPC and 36% using services during bereavement. Dionne-Odom and colleagues described a similar rate of approximately 30% of FCs of advanced cancer patients having accessed support services, but with an additional 25% of FCs who would have been interested [30]. In a study by Aoun et al., FCs also indicated that informal support by family or friends was the most frequently used form of support, followed by services offered by undertakers [32], which we did not include as an answer option. In our study, the most frequently used psychosocial support services over time were consistently psychological, spiritual and social counseling. Only approximately one-third of the FCs made use of different bereavement care programs offered by the study wards. This finding might indicate that grief work represents a very personal matter, resulting in less interest in professional than informal bereavement support. It is possible that FCs also do not feel that they are the target users of available supportive care offers. At both points in time, non-users of support services mostly indicated that they had not accessed services due to sufficient informal support and a subjective lack of need for support (prior to SIPC: 71–84%, during bereavement: 59–78%). In comparison, structural problems, such as lack of time, lack of knowledge about services and services being too far away, were reported less often (prior to SIPC: 20–45%, during bereavement: 24–28%). Less is known about factors influencing the acceptance of bereavement support, but psychosocial support during the patient’s disease seems to be used more frequently by FCs with higher psychological burden, anxiety, and depression; lower preparedness; more perceived helpfulness; and higher unmet supportive needs [11, 19, 30, 31].

## Limitations

Our study has some limitations. First, the results need to be carefully interpreted in terms of possible selection bias and limitations of the generalizability of the results. Of all 693 patients admitted to the SIPC wards during the recruitment phase, the FCs of 143 of them (21%) had to be excluded for patient-related reasons, most often due to close proximity of the patient’s death. For a further 272 of 693 patients (39%), their FCs were not eligible due to caregiver-related reasons or FCs’ refusal of study participation, mostly due to high psychological burden. It is possible that non-participants may have been struggling even more, and hence, supportive care needs may be underestimated. However, research on these vulnerable FCs is sensitive, and FCs’ integrity must be accommodated for ethical reasons [33]. The strengths of our study include the longitudinal design, consecutive recruitment strategy, and systematic documentation of non-participants and non-respondents.

## Conclusion

In conclusion, FCs of advanced cancer patients identified a high number of very or extremely important needs at the beginning of SIPC and near the patient’s death. Regarding specific needs, the importance of needs remained relatively stable over time, while changes in satisfaction with fulfillment of FCs’ needs were observed in some areas. Approximately one-fourth of at least very important needs remained unmet for the majority of FCs at both assessments. Thus, a frequent reassessment of FC needs as well as the establishment of specific programs and services to meet the identified and unmet needs of FCs throughout the caregiving process is required. The identified factors associated with FC needs, including degree of satisfaction with care, extent of informal social support, and FC appointment as substitute decision-maker, can be used to help allocate healthcare resources to those with intensified need for support. However, only approximately one-third of FCs used information and support services prior to and during SIPC as well as in the bereavement phase. The main reason was that FCs relied on sufficient informal support and did not perceive themselves to need such services. Thus, FCs need to be better informed about the scope and beneficial effects of professional support services and how these services can complement informal support and vice versa. Furthermore, the future situation of FCs of advanced cancer patients could be improved through the provision of services tailored to FC needs as well as FC-specific written or audio-visual information, e.g., in the format of brochures, online tools, or videos. Through these strategies, FCs will be assured that their specific needs are of interest to healthcare providers; additionally, it is inevitable that FCs will need to be assured that supportive care needs are often experienced during caregiving and that many of these needs can be adequately addressed by professionals. To enhance the delivery of clinically relevant and effective supportive interventions, attention should be paid to barriers that may impede the utilization of FC-directed supportive care in the conceptualization, development, and implementation of such care.

## Data Availability

The authors have full control over the primary data. The data are analyzed in this study are housed at the Palliative Care Unit, Department of Oncology, Hematology and BMT, University Medical Center Hamburg-Eppendorf, Martinistrasse 52, 20,246 Hamburg, Germany.
